# *De novo* assembly, annotation, and characterization of the whole brain transcriptome of male and female Syrian hamsters

**DOI:** 10.1038/srep40472

**Published:** 2017-01-10

**Authors:** Katharine E. McCann, David M. Sinkiewicz, Alisa Norvelle, Kim L. Huhman

**Affiliations:** 1 Neuroscience Institute Georgia State University, 161 Jesse Hill Jr. Drive Atlanta, Georgia 30303, USA.

## Abstract

Hamsters are an ideal animal model for a variety of biomedical research areas such as cancer, virology, circadian rhythms, and behavioural neuroscience. The use of hamsters has declined, however, most likely due to the dearth of genetic tools available for these animals. Our laboratory uses hamsters to study acute social stress, and we are beginning to investigate the genetic mechanisms subserving defeat-induced behavioural change. We have been limited, however, by the lack of genetic resources available for hamsters. In this study, we sequenced the brain transcriptome of male and female Syrian hamsters to generate the necessary resources to continue our research. We completed a *de novo* assembly and after assembly optimization, there were 113,329 transcripts representing 14,530 unique genes. This study is the first to characterize transcript expression in both female *and* male hamster brains and offers invaluable information to promote understanding of a host of important biomedical research questions for which hamsters are an excellent model.

Syrian hamsters (*Mesocricetus auratus*) have been used in biomedical research for decades because they are uniquely suited for the study of a wide variety of behaviours and diseases. In recent years, however, the use of hamsters has declined^1^. A PubMed search of ‘Syrian hamster’ yields 2,280 publications before 1995, 856 publications from 1995–2004, and only 463 publications from 2005–2015. This decline is likely due to the advancement in genetic and molecular tools for other rodents, namely mice, and is not due to a reduction in the utility of hamsters in biomedical research. For example, hamsters provide an excellent model with which to study many types of cancer^2^,^3^, a variety of tumours^4^,^5^, and even pathogens such as Ebola virus^6^,^7^. Hormone release from the hypothalamic-pituitary-adrenal (HPA) axis, the so-called stress axis, in humans is more similar to that of hamsters than it is to that of other rodents, making hamsters a valuable model for studying behavioural and neurochemical responses to stress^8^,^9^,^10^,^11^,^12^. In addition, hamsters display robust circadian rhythms^13^,^14^, which make them an ideal subject for the study of the neurobiological basis of circadian rhythmicity. Finally, both male and female hamsters display a rich array of social and communicative behaviours, including intraspecific aggression and striking behavioural responses to social defeat stress^15^,^16^,^17^,^18^,^19^, allowing for the study of sex differences in a wide variety of endpoints using this species.

Historically, the vast majority of scientific research has used primarily male subjects, whether the study involved non-humans or humans. This has certainly been the case in the majority of neuroscience research using rodent models^20^. This bias towards males has historically been attributed to the complexity and variability introduced by working with females that have pronounced fluctuations in hormonal state, but it is also the case that, among mammals, some behaviours are not prominently produced by females (e.g., territorial aggression). Female rats and mice, for example, rarely produce any aggression outside of maternal defence of pups^21^. It is clearly the case, however, that female humans can be highly aggressive even outside of defence of offspring, thus rats and mice may not represent the best choice with which to model human agonistic behaviour. Female hamsters, on the other hand, readily display a range of social and agonistic behaviours toward male and female conspecifics^18^,^22^,^23^,^24^,^25^, presenting the opportunity to study social behaviour in both sexes rather than trying to generalize findings from males to females.

Our lab has established a model of social stress-induced behavioural change in Syrian hamsters that we have termed conditioned defeat. Conditioned defeat is the dramatic shift from territorial aggression to submission and social avoidance that is exhibited by both males and females after losing even a *single* agonistic encounter^9^,^18^,^26^. We have begun to examine the genetic and epigenetic markers of conditioned defeat but have been limited in this pursuit by a lack of specific probes and primers that are selective for hamster gene sequences. Thus, to advance the tools with which to investigate potential genetic mechanisms leading to conditioned defeat as well as to sexual dimorphisms in social behaviour, we sequenced the entire brain transcriptome of males and females. Here, we provide a detailed analysis of the brain transcriptome of male and female hamsters. This novel information about transcript expression in hamster brain will be of wide utility in a variety of fields that currently use hamsters as well as to fields that currently rely on mouse models of illnesses or behaviours for which hamsters would be ideal subjects.

## Results and Discussion

### Sample quality and description of raw reads

All RNA samples were measured with the Agilent Bioanalyzer before sequencing. The RNA integrity numbers (a measure of sample quality) of all samples were good, falling between 7–8 (maximum value of 10), and all above the recommended cut-off of 6. Table 1 shows the RNA quality and concentration of each sample. Final raw sequence data was run through a quality assurance test (FastQC) to ensure minimal bias in sequencing and to confirm quality of starting library material. This test provides confidence in the quality of the sequence output before proceeding to assembly and annotation. Per base sequence quality scores all fell in the “very good” range (Phread score above 28) giving us the confidence to move forward with transcriptome assembly.

### Transcriptome assembly

We assembled the Syrian hamster brain transcriptome using *de novo* techniques because, while there is a partially annotated Syrian hamster genome available (NCBI NW_004801604.1, APMT 00000000.1), we were unable to reliably use this for a genome-guided assembly for several reasons. First, the genome currently available was sequenced from a single female hamster, thus eliminating the sequences of any Y-linked genes. One of the goals of this project was to develop tools to be able to directly compare males and females, so having Y-linked sequences would not only provide a positive control when looking at sex differences but would also lead to a more complete and representative transcriptome. In addition, the incomplete annotation of the current hamster genome leads to a number of problems when trying to build a transcriptome. The software currently available for building genome-guided assemblies assumes complete, or near-complete, annotation, and therefore returns error messages for any sequence that is not already annotated. Thus, we moved forward with a *de novo* assembly for more accurate and complete results.

The *de novo* assembly using Trinity revealed 1,002,166 total Trinity ‘genes’ and 1,147,108 transcripts from 973,648,406 total assembled bases. The average contig, or presumptive transcript, was 848.79 bases (median 440) with a percent GC content of 45.62. After completing the *de novo* assembly, raw reads were aligned back to the assembly. Proper pairs (both left and right reads aligned to same contig) accounted for 80.83% (539,735,450) of the 667,738,987 total aligned reads. Of the remaining pairs, left-only reads accounted for 9.68% (64,655,456) and right-only for 7.85% (52,410,243). Improper pairs, in which left and right reads align but to different contigs due to fragmentation, accounted for only 1.64% (10,937,838) of the total reads. These data provide an excellent starting point with which to build a usable transcriptomic database for Syrian hamster brains.

### Assembly optimization and annotation

**Figure 1:**
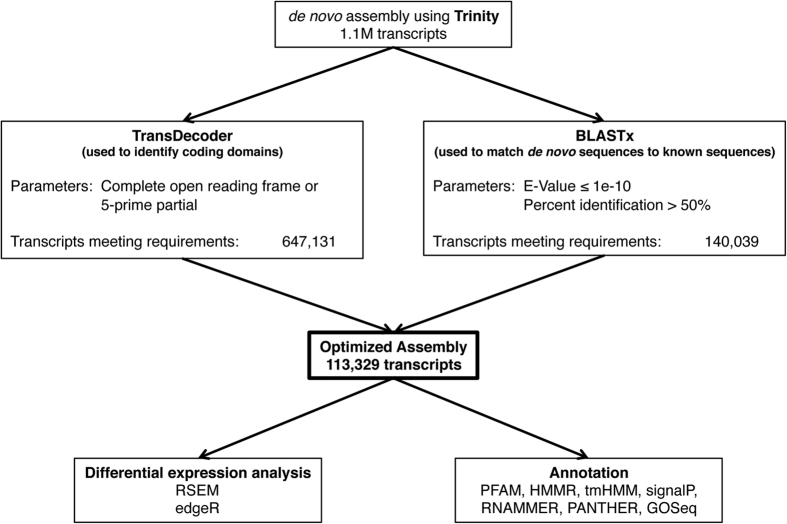
Schematic of *de novo* assembly optimization and analysis. After initial *de novo* assembly using Trinity, we optimized the assembly using several programs to omit falsely assembled sequences or sequences that were not likely to code for an actual gene. After optimization, we used RSEM to generate expected counts of each transcript from the raw reads and used those reads to calculate differential expression between males and females using edgeR. Annotation of the optimized assembly was completed using a series of annotation steps, PANTHER, and GOSeq.

Trinity ‘genes’ are transcripts that may or may not code for a specific gene. Trinity *de novo* sequencing builds transcripts from sequence patterns that are *likely* to code for a gene. Without a genome to guide the assembly, some guesswork is involved in assembling the bases into known sequences. Thus, the approximation of the *de novo* assembly calls for several additional parameters to be put in place to build a more confident and usable transcriptome database. In order to gain confidence in our assembly and to minimize false positives as well as artificial sequences created by the *de novo* assembly, we ran a number of programs (see Methods) to optimize the assembly into an accurate representation of transcripts present in Syrian hamster brain, as done previously with other *de novo* assemblies in several fish and rodent species^27^,^28^,^29^,^30^,^31^. See Fig. 1 for a schematic of the assembly optimization process.

First, TransDecoder was run to determine the number of probable coding sequences within the assembly. Complete coding sequences accounted for 456,234 of the total number of open-reading frames (790,773). There were 108,213 3′-partial, 190,897 5′-partial, and 35,429 internal sequences. The sequencing protocol had a 3′ bias, thus we included all transcripts with 5′-partial and complete coding sequences for the initial assembly optimization (647,131), as these transcripts were most likely to represent expressed genes^32^. We also filtered the assembly using data obtained from BLASTx using the Uniprot-rodent database (1/21/16) to ensure that all transcripts matched a known rodent sequence. BLASTx returned 1,219,140 matches, however many of these were at very low confidence parameters, thus only those with an E-value of ≤1e-10 and a percent identification match of ≥50 were included (140,039). These stringent parameters provide enhanced confidence in the quality of our optimized and annotated transcriptome^28^,^29^. Finally, we combined the output from TransDecoder and BLASTx, which left 113,329 transcripts meeting all the above stated criteria. An additional 27 transcripts were identified as containing a vector sequence during submission to the NCBI database and were removed from the final assembly. While this reduction process may have eliminated some sequences that represent true genes within hamster brain, these steps were necessary in order to eliminate a large number of false positives that can occur in *de novo* sequencing. Furthermore, BUSCO analysis revealed that 89% of the highly conserved sequences among vertebrates were present in the optimized assembly (2695 out of 3023), while 92% of the conserved genes across all eukaryotes were present (396 out of 429). These data also provide enhanced confidence in the quality and completeness of the optimized brain transcriptome.

**Figure 2:**
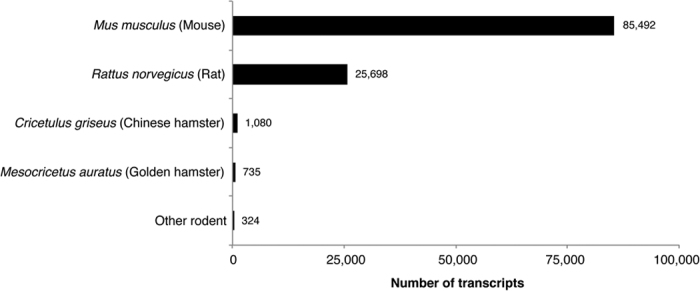
Number of transcripts matching specific rodent species. The majority of transcripts in the optimized assembly matched *Mus musculus* as the top hit during annotation. Over 700 transcripts matched the partially annotated *Mesocricetus auratus* genome, suggesting a strong alignment of the *de novo* assembly with the available hamster genomic sequences.

We used the rodent database from Uniprot in order to maximize the number of transcripts in our assembly that matched a known sequence. Almost all of the transcripts matched *Mus musculus* (mouse) (85,492) and/or *Rattus norvegicus* (rat) (25,698), while 735 transcripts matched *Mesocricetus auratus* (golden hamster) as the top hit (Fig. 2). This is not surprising considering that the mouse genome is the most highly curated rodent genome available. Of the 113,329 individual transcripts in the optimized assembly, there were only 14,530 unique gene identifiers from BLAST, suggesting that there are multiple isoforms of some genes present in the assembly. This is consistent with data in mice and humans showing that there are approximately 17,000–25,000 genes in their respective genomes, with at least 10x the number of transcripts^33^,^34^,^35^,^36^. Of the 735 transcripts in the optimized transcriptome that matched *M. auratus*, there were 155 unique gene identifiers from BLAST. There are only 274 reviewed and annotated Syrian hamster genes in the Uniprot database, and more than half of those sequences match sequences from our *de novo* transcriptome assembly. Furthermore, many of the *de novo* sequences match multiple species in BLAST and only the top hit is recorded for this annotation. Therefore, it is likely that many more transcripts matched *M. auratus* but also matched another rodent (e.g., *M. musculus* or *R. norvegicus*) with an equal or higher score. Overall, the close alignment with the partially annotated hamster genome further validates our *de novo* assembly.

### Transcript expression analyses

Using expected read counts from RSEM, we first compiled a matrix to determine which transcripts were most highly expressed in Syrian hamster brain. The genes represented by these transcripts are shown in Table 2 and, not surprisingly, represent genes that are highly expressed in brain tissue of other species. For example, prosaposin is important for nervous system development and maintenance and microtubule-associated protein 1a is critical for neurogenesis and is found at its highest levels in brain tissue of rodents and humans^37^,^38^. Furthermore, several of the top expressed transcripts are nervous system-specific, including two of the top five expressed transcripts that are myelin-related (myelin proteolipid protein and myelin basic protein), as well as neuronal membrane glycoprotein M6-a.

**Figure 3:**
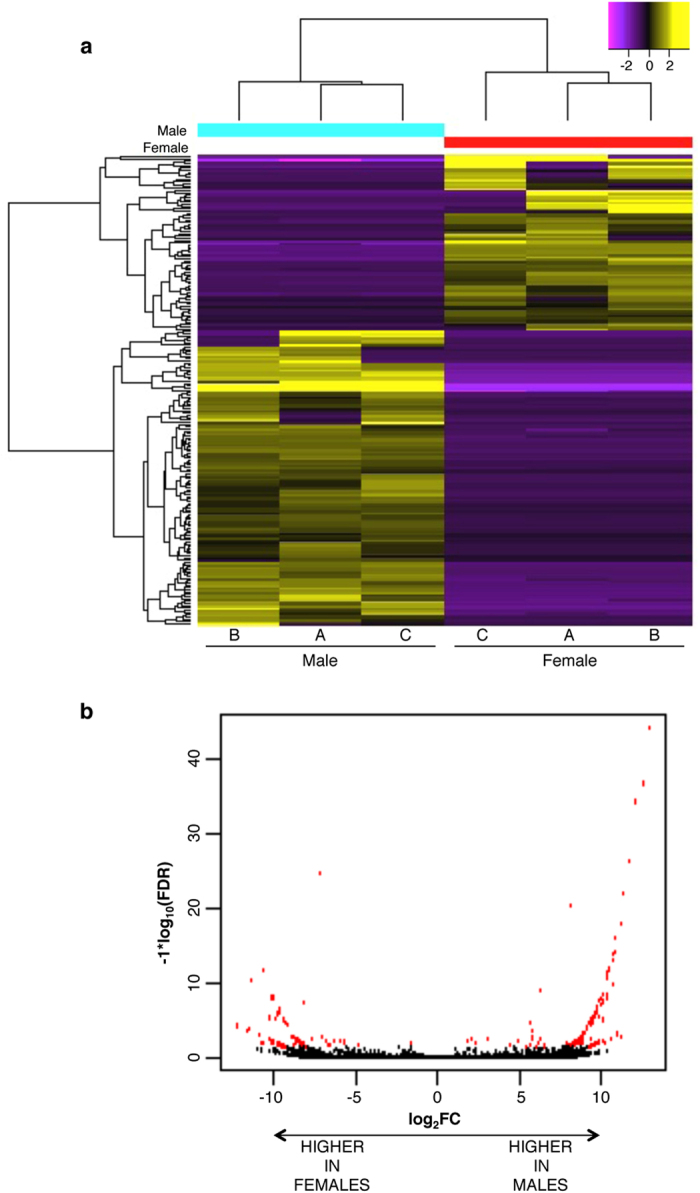
Visualization of differential expression between male and female hamster brain. (**a**) Heatmap showing the 207 differentially expressed transcripts between males and females. The left side of the heatmap indicates how the transcripts group together and fold change is shown by colour (yellow designating positive fold change, purple designating negative fold change). More transcripts (130) were higher in males than were higher in females (77). (**b**) Volcano plot of the transcripts expressed in hamster brain. Red indicates a significant difference in expression between males and females (FDR < 0.05).

We next completed differential expression analysis on the optimized transcriptome to determine what transcripts, if any, were differentially expressed in male and female brains. Excluding transcripts that did not meet the minimum expression cut off (see Methods), 207 transcripts were differentially expressed in the whole brain, the majority of which were higher in males compared with females (130 higher in males, 77 higher in females) (Fig. 3). Some of the differentially expressed transcripts (DETs) matched the same BLAST entry, suggesting that there may be differential regulation of multiple isoforms of these genes. The full list of genes represented by the DETs can be found in Supplemental Table S1.

There are several important considerations regarding DETs that should be addressed. First, they are presented here based on which sex had higher expression. It should be noted that the differential expression could, in fact, be the result of a decrease in expression of the opposite sex or a combination of an increase in one and a decrease in the other. Second, 207 is a reasonable number to expect for overall sex differences in whole brain based on data from both humans and drosophila^39^,^40^, however this number can vary greatly depending on the statistical test and parameters used. Here, we use a stringent analysis previously used in other *de novo* assemblies and one recommended by the Trinity package^28^,^41^. Lastly, the differences reported here are representative of the entire brain, thus some sexually dimorphic genes may not be represented in our dataset due to differential regulation in different brain regions that may act to counterbalance or eliminate overall differences in expression. It is interesting to note that some of the DETs from the transcriptome represent genes that have been shown to be sexually dimorphic in other species. For example, one isoform of tolloid-like protein 1 (Tll1) was more highly expressed in females, while another isoform was higher in males. Tll1 has been linked to sex differences in behavioural response to stress in mice^42^ and, based on the current data it may be of interest to further define the role of specific isoforms of this gene in both males and females. The consistency of sexual dimorphism in our hamster transcriptome compared with other species indicates that this *de novo* assembly will be a powerful toolkit for future use in hypothesis-driven investigation of gene expression in male and female hamsters.

### Functional annotation and gene ontology (GO) analysis

**Figure 4:**
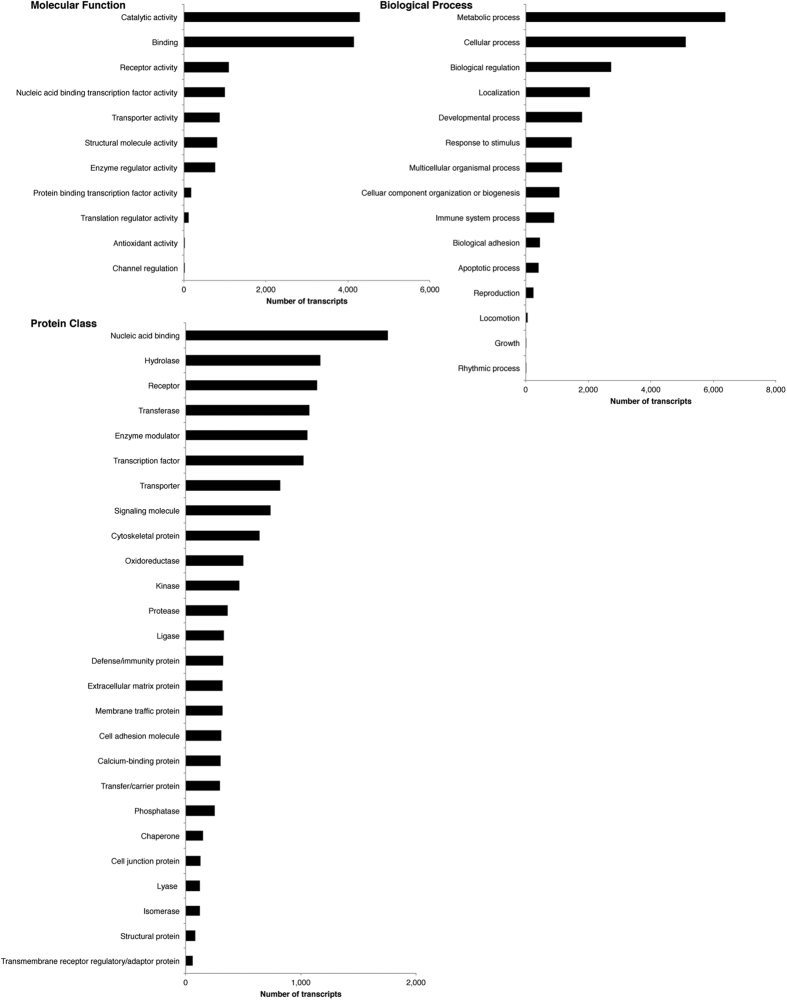
Highest represented gene ontology terms from the optimized whole brain transcriptome. We used PANTHER analysis to match the 14,530 unique genes in the optimized transcriptome to gene ontology terms for functional annotation of the assembly. These are the most represented functions in Syrian hamster brain.

**Figure 5:**
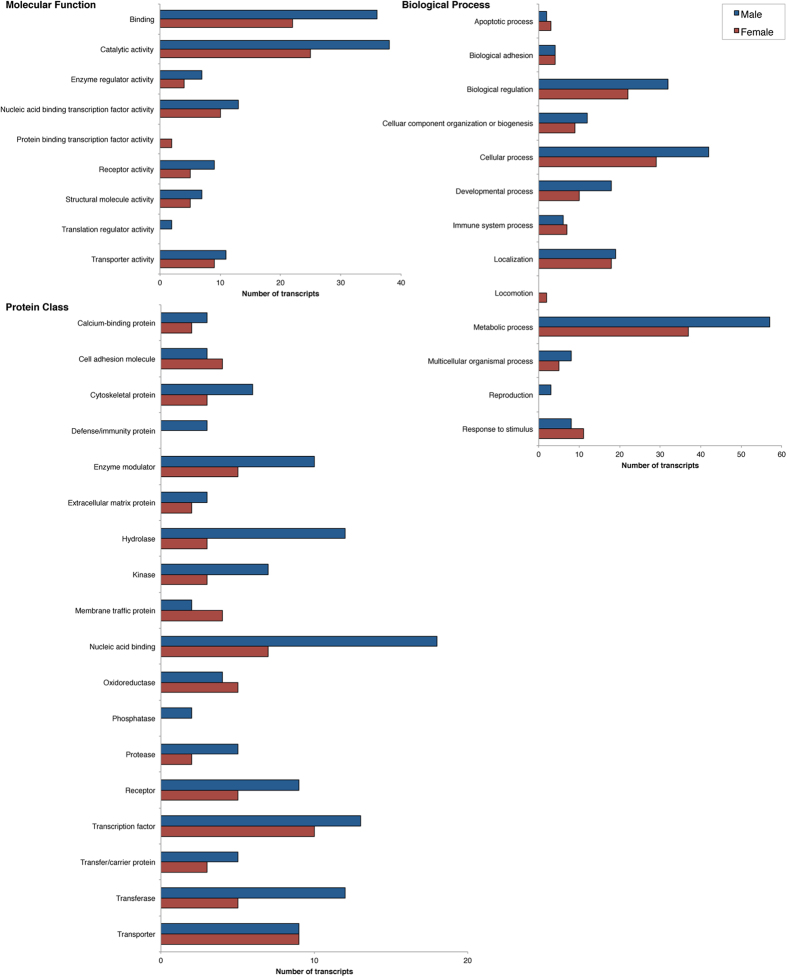
Highest represented gene ontology terms in the subsets of differentially expressed genes. Highest represented gene ontology terms from PANTHER for the 130 genes more highly expressed in males (blue) and the 77 genes more highly expressed in females (red) in Syrian hamster brain.

Annotation of the optimized assembly was completed using the steps outlined in Methods. The results from the assembly annotation are shown in Table 3. In order to complete functional annotation of the full brain transcriptome, we next filtered the annotated assembly through PANTHER analysis to determine which GO terms were highly represented in the optimized brain transcriptome. The top hits for each classification (molecular function, biological process, protein class) are presented in Fig. 4. Next, we examined the subsets of DETs to determine if any specific GO terms differed in their representation in these transcripts as compared with the complete transcriptome. The highest represented terms for each classification in males and females are presented in Fig. 5. Catalytic activity and binding were the most represented molecular functions in the full assembly as well as in the subsets of DETs. Likewise, the highest number of transcript matches for biological processes were cellular and metabolic processes.

Each category represented in Figs 4 and 5 has subcategories into which the transcripts can be further classified, and several interesting trends emerge when comparing the DETs. For example, the vast majority of transcripts associated with Localization in males (85.1%) and females (81.9%) matched the highest categories for the whole brain, including Vesicle, Protein, Ion, and Lipid Transport (81.8%). In addition, the majority of Receptors classified in the optimized brain transcriptome represented G-protein Coupled Receptor Activity (42.5%) but none of the transcripts that were differentially expressed between males and females were classified by this subcategory. In fact, Glutamate Receptor Activity was the only subcategory of Receptor represented in the transcripts with differential expression (higher in females). These functional classifications of the DETs may help to identify more precise targets for understanding sex differences in behaviour and future studies can explore these possibilities.

**Figure 6:**
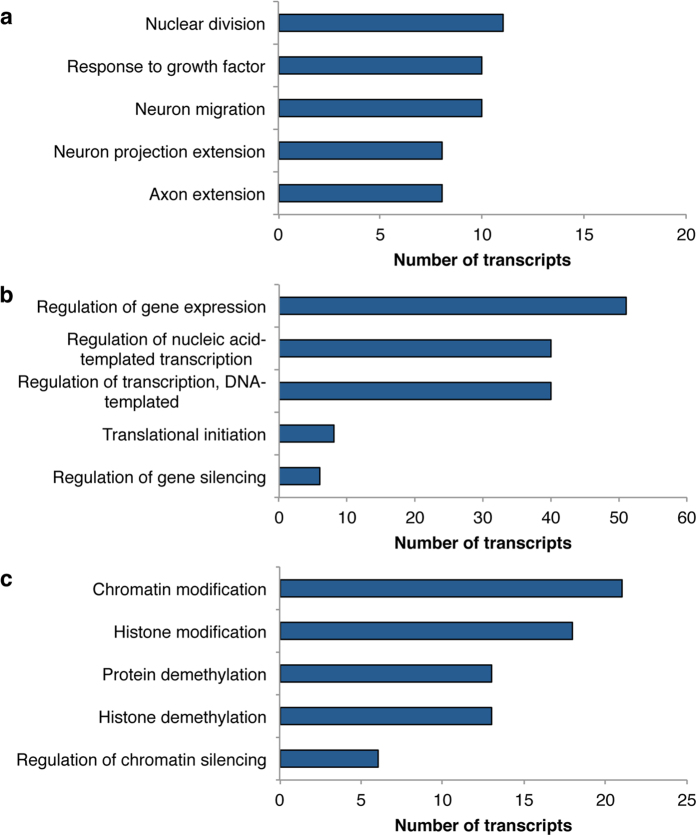
Enriched gene ontology terms from GOSeq analysis. GOSeq revealed 142 gene ontology terms that were enriched in males compared with females, the majority of which were categorized as Biological Processes. Subsets of these terms are shown here and involve processes related to (**a**) growth, (**b**) gene expression, and (**c**) epigenetic modification.

Finally, an enrichment analysis using GOSeq revealed 142 GO terms that were enriched in DETs that were more highly expressed in males. The majority of these terms (i.e., 100) were in the category of biological process and involved gene expression, epigenetic modification, and growth. A subset of these terms is highlighted in Fig. 6 and a full list can be viewed in Supplemental Table S2.

## Conclusions

These data represent the first comprehensive report of the Syrian hamster brain transcriptome and the first time that transcripts of both male and female hamsters have been sequenced and analysed. The differential expression analyses presented here between male and female baseline expression are not meant to provide a detailed analysis of sex differences in the brain but rather to provide a good starting point for analysing potential genetic and epigenetic mechanisms underlying sex differences in behaviour. Our lab is currently investigating site-specific sex differences in transcript expression in the brain using the tools developed here. Ultimately, the sequences obtained from this project will permit those conducting biomedical research to use Syrian hamsters when appropriate and to design custom primers and probes using hamster-specific sequences to answer important molecular and genetic questions.

## Methods

### Animals and tissue collection

Six adult male and six adult female Syrian hamsters were obtained from Charles River Laboratories (Danvers, MA). Animals were approximately 10 weeks old upon arrival and weighed between 120–130 g. Subjects were singly housed for at least 2 weeks and handled daily. During handling, oestrous cycles of females were monitored for at least two cycles via vaginal swabs to confirm oestrous cycle stage and stability. For brain collection, animals were anesthetized via isoflurane exposure and then decapitated. All females were sacrificed on Dioestrus 2 to minimize variation in gene expression based on day of the oestrous cycle. This day of the cycle was chosen because we most often test female behaviour on Dioestrus 2 in our laboratory^43^. An equal number of males were sacrificed at the same time as the females. After decapitation, whole brains were rapidly extracted, frozen immediately in isopentane on dry ice, and stored at −80 °C until processing. All procedures and protocols were approved by the Georgia State University Institutional Animal Care and Use Committee and are in accordance with the standards outlined in the National Institutes of Health Guide for Care and Use of Laboratory Animals.

### RNA extraction

Two brains from same-sex animals were pooled together for each RNA extraction in order to minimize the effect of individual variability. This sample size supplies sufficient power for downstream analyses while minimizing the total number of animals sacrificed^44^,^45^. We used Trizol (Life Technologies, Grand Island, NY) for extractions, following a modified version of the manufacturer’s protocol. In brief, frozen brains were cut into large pieces and placed in 50 mL conical tubes on ice. Brains were homogenized on ice with 20 mL Trizol. After full homogenization, the sample was allowed to settle at room temperature for 5 min. The homogenate was then mixed with 4 mL of chloroform, allowed to stand at room temperature for 2–3 min and centrifuged at 5,250 × g for 45 min at 4 °C to separate the phases. The aqueous RNA phase was removed and dispensed into a new conical tube. 200 μL/mL of chloroform was added to the aqueous phase, mixed well, allowed to stand 2–3 min, and then centrifuged at 12,000 × g for 10 min at 4 °C. For enhanced visualization of the pellet, 3 μL/mL of GlycoBlue (Life Technologies, Grand Island, NY) was added and mixed gently. For RNA precipitation, 500 μL/mL of 100% isopropanol was added, mixed gently and allowed to stand at room temperature for 10 min. To obtain an RNA pellet, the solution was centrifuged at 12,000 × g for 20 min at 4 °C. The remaining liquid was carefully removed and the pellet was washed twice in 75% RNase-free ethanol and centrifuged at 7,500 × g for 5 min at 4 °C. The pellet was allowed to air dry for approximately 5 min and was then re-suspended in 125 μL of ultrapure water and immediately stored at −80 °C.

### RNA quality assurance and RNA sequencing

RNA quality was assessed using the Agilent RNA 6000 Nano Kit (Agilent Technologies, Santa Clara, CA) on the Agilent Bioanalyzer, following the manufacturer’s instructions. RNA integrity numbers and concentration (ng/μl) were recorded and sent with the samples for sequencing. Samples (n = 6) were sent on dry ice to Beckman Coulter Genomics (Danvers, MA) for Illumina Automated RNA sequencing and were sequenced in paired-end 100 bp reads, averaging 110 M reads per sample. While it is true that 110 M reads may not allow for the identification of the entire transcriptome (e.g., microRNAs, non-coding RNAs), it should identify without any issue the mRNA landscape of male and female brain tissue^46^.

### Transcriptome assembly and optimization

In order to produce a comprehensive brain transcriptome, we completed a *de novo* transcriptome assembly with Trinity (https://github.com/trinityrnaseq/trinityrnaseq)^47^,^48^ using the jaccard clip parameter to minimize potential fusion transcripts. All data were acquired using the computing resources at Georgia State University^49^. After assembly, TransDecoder (https://transdecoder.github.io/)^48^ was used to identify coding domain sequences with a minimum cut-off of 50 amino acids^28^. Assembled transcripts were also run through NCBI’s BLASTx (National Center for Biotechnology Information’s Basic Local Alignment Search Tool, http://blast.ncbi.nlm.nih.gov/Blast.cgi)^50^ using the Uniprot-rodent database from January 21, 2016 (http://www.uniprot.org/)^51^ to match *de novo* sequences to known genes.

Annotation of the assembly was accomplished using a series of annotation steps, including NCBI’s BLAST to match sequences to known genes, Pfam^52^ and HMMR^53^ to identify protein domains, tmHMM^54^ to predict transmembrane regions, signalP^55^ to predict signal peptides, and RNAMMER^56^ to identify rRNA transcripts. Finally, we compared our annotated assembly to a database of highly conserved orthologs using the BUSCO (Benchmarking Universal Single Copy Orthologs, http://busco.ezlab.org/) database to determine the completeness of our optimized assembly^31^,^57^.

We further identified gene ontology terms associated with our annotated transcripts using PANTHER (Protein Analysis Through Evolutionary Relationships, http://pantherdb.org/)^58^,^59^,^60^,^61^. We compared all genes using *Mus musculus* as the reference organism in PANTHER and identified the molecular functions, biological processes, and protein classes associated with the fully annotated transcriptome and the subsets of DETs, described below. GOSeq was used to perform the enrichment analysis on the differentially expressed transcripts^62^.

### Differential expression analysis

Differential transcript expression in male and female hamster brains was calculated using an exact test in the Bioconductor R package (https://www.r-project.org/), edgeR (Empirical Analysis of Digital Gene Expression Data in R, https://bioconductor.org/packages/release/bioc/html/edgeR.html)^63^,^64^. We used RSEM (RNA-Seq by Expectation-Maximization, http://deweylab.github.io/RSEM/)^65^ to generate read counts for the optimized assembled transcriptome to input into edgeR. EdgeR normalizes raw input data using a trimmed mean of M-values (TMM) and transcripts with artificially low counts (<1 count across all samples) after normalization were excluded before differential expression analysis was completed. Transcripts were considered to significantly differ in expression between males and females if the false discovery rate (FDR) was <0.05. All transcripts that met this criteria had a log_2_ fold change of <−2 or >2, with the exception of two transcripts (−1.67, 1.86). These stringent parameters used as the cut-off for considering transcripts to be differentially expressed greatly minimizes the possibility of false positives.

## Additional Information

**Accession codes:** This Transcriptome Shotgun Assembly project has been deposited at DDBJ/EMBL/GenBank under the accession GEMX00000000 (BioProject PRJNA320732). The version described in this paper is the first version, GEMX01000000.

**How to cite this article**: McCann, K. E. *et al*. *De novo* assembly, annotation, and characterization of the whole brain transcriptome of male and female Syrian hamsters. *Sci. Rep.* **7**, 40472; doi: 10.1038/srep40472 (2017).

**Publisher's note:** Springer Nature remains neutral with regard to jurisdictional claims in published maps and institutional affiliations.

## Supplementary Material

Supplementary Information

## Figures and Tables

**Table 1:** Individual sample quality and concentration of each sample pool used for sequencing.

Sample Pool	RNA integrity number (RIN)	Concentration (ng/μl)
Female A	7.7	802
Female B	7.3	1286
Female C	7.3	848
Male A	7.4	1231
Male B	7.7	915
Male C	7.4	992

**Table 2:** Most highly expressed genes.

Gene ID	Gene	Uniprot ID
Nlrc3	Protein NLRC3	NLRC3_MOUSE
Plp1	Myelin proteolipid protein	MYPR_RAT
Scd2	Acyl-CoA desaturase 2	ACOD2_RAT
Hspa8	Heatshock cognate 71 kDa	HSP7C_RAT
Mbp	Myelin basic protein	MBP_MOUSE
Eef1a1	Elongation factor 1-alpha-1	EF1A1_RAT
Gapdh	Glyceraldehyde-3-phosphate dehydrogenase	G3P_CRIGR
Ywhag	14-3-3 protein gamma	1433G_RAT
Hsp90aa1	Heat shock protein HSP 90-alpha	HS90A_MOUSE
Sptbn1	Spectrin beta chain, non-erythrocytic 1	SPTB2_MOUSE
Atp5b	ATP synthase subunit beta, mitochondrial	ATPB_RAT
Glul	Glutamine synthase	GLNA_ACOCA
Aldoa	Fructose-bisphosphate aldolase A	ALDOA_RAT
Camk2n1	Calcium/calmodulin-dependent protein kinase II inhibitor 1	CK2N1_RAT
Atp2a2	Sarcoplasmic/endoplasmic reticulum calcium ATPase 2	AT2A2_MOUSE
Snrpn	Small nuclear ribonucleoprotein-associated protein N	RSMN_RAT
Psap	Prosaposin	SAP_RAT
Map1a	Microtubule-associated protein 1A	MAP1A_MOUSE
Serinc1	Serine incorporator 1	SERC1_RAT
Gpm6a	Neuronal membrane glycoprotein M6-a	GPM6A_RAT

Top 20 genes that are the most highly expressed in Syrian hamster brain (both males and females).

**Table 3:** Annotation of assembly.

Annotation Step	Purpose	Number of transcripts
RNAMMER	Identify rRNA transcripts	44
PFAM	Identify protein domains	103,916
SignalP	Predict signal peptides	29,004
tmHMM	Predict transmembrane regions	36,978

Number of transcripts represented in each step of the annotation process.
