# Supplementary material for: De novo assembly, annotation, and characterization of the whole brain transcriptome of male and female Syrian hamsters
Source: Sci Rep. 2017 Jan 10;7:40472. doi: 10.1038/srep40472 (PMC5223125; doi:10.1038/srep40472)
Supplement: Supplementary Information [file srep40472-s1.pdf]

***De novo* assembly, annotation, and characterization of the whole brain transcriptome of male and female Syrian hamsters – Supplemental Information**

KE McCann, DM Sinkiewicz, A Norvelle, KL Huhman

Supplemental Table S1 Differential expression in male and female whole brain

A comprehensive list of the transcripts that were differentially expressed in whole brain of male and female hamsters. Expression indicates in which sex the gene was more highly expressed. If both sexes are indicated, different isoforms of the same gene were differentially expressed in males and females. Transcripts with more than one isoform that were differentially expressed in the same direction are indicated by an *. The two isoforms with the largest log_2 fold change are listed here. (Log_2CPM = log_2 counts per million; Log_2FC = log_2 fold change; FDR = false discovery rate)

Gene ID	Gene	Uniprot ID	Expression	Log_2CPM	Log_2FC	FDR
**Abcb7**	ATP-binding cassette sub-family B member 7, mitochondrial	ABCB7_RAT	FEMALE	1.8682	-9.8993	8.10e-06
**Abcb10**	ATP-binding cassette sub-family B member 10, mitochondrial	ABCBA_MOUSE	FEMALE	0.5178	-8.4718	0.0097
**Adgra1**	Adhesion G protein-coupled receptor A1	AGRA1_MOUSE	FEMALE	0.798	-8.7755	0.021
**Anapc1**	Anaphase-promoting complex subunit 1	APC1_MOUSE	MALE	1.6556	9.6833	0.0155
**Apex1**	DNA-(apurinic or apyrimidinic site) lyase	APEX1_MOUSE	FEMALE	0.8734	-8.8556	0.0016
**Asap3**	Arf-GAP with SH3 domain, ANK repeat and PH domain-containing protein 3	ASAP3_MOUSE	MALE	0.3546	8.3007	0.0254
**Atp13a3**	Probable cation-transporting ATPase 13A3	AT133_MOUSE	FEMALE*	3.4765<br>2.1969	-11.5394<br>-10.2374	0.0002<br>0.0043
**Atp2b1**	Plasma membrane calcium-transporting ATPase 1	AT2B1_RAT	MALE	1.3529	9.3655	0.0278
**Atp2c1**	Calcium-transporting ATPase type 2c member 1	AT2C1_RAT	MALE	0.5279	8.49	0.0056
**Bcor**	BCL-6 corepressor	BCOR_MOUSE	MALE	1.7008	9.7289	1.74e-07
**Bmpr1b**	Bone morphogenetic protein receptor type-1B	BMR1B_MOUSE	MALE	1.6322	9.6567	2.38e-05
**Brd8**	Bromodomain-containing protein 8	BRD8_MOUSE	MALE	1.4198	9.437	5.49e-06
**Btf3l4**	Transcription factor BTF3 homolog 4	BT3L4_MOUSE	FEMALE	1.3263	-9.3357	0.0335
**C1ql3**	Complement C1q-like protein 3	C1QL3_MOUSE	FEMALE	2.8039	-10.8576	0.001
**Ccdc186**	Coiled-coil domain-containing protein 186	CC186_MOUSE	MALE	1.5801	9.6025	0.0161
**Ccm2**	Cerebral cavernous malformations protein 2 homolog	CCM2_MOUSE	MALE	4.4286	12.4994	2.10e-37
**Ccnt1**	Cyclin-T1	CCNT1_MOUSE	FEMALE	0.1306	-8.0454	0.0447

Gene ID	Gene	Uniprot ID	Expression	Log_2CPM	Log_2FC	FDR
**Ccs**	Copper chaperone for superoxide dismutase	CCS_RAT	FEMALE	0.7685	-6.8224	0.0056
**Cdr2l**	Cerebellar degeneration-related protein 2-like	CDR2L_MOUSE	FEMALE	0.5228	-6.5572	0.0278
**Cep68**	Centrosomal protein of 68 kDa	CEP68_MOUSE	FEMALE	0.5788	-8.5387	0.0047
**Cfh**	Complement factor H	CFAH_MOUSE	MALE	2.8678	10.9244	0.0007
**Csgalnact1**	Chondroitin sulfate N-acetylgalactosaminyltransferase 1	CGAT1_MOUSE	MALE	0.8869	8.8755	0.0009
**Chd1**	Chromodomain-helicase-DNA-binding protein 1	CHD1_MOUSE	MALE	0.7058	8.6822	0.0022
**Chd5**	Chromodomain-helicase-DNA-binding protein 5	CHD5_MOUSE	FEMALE<br>MALE	3.2977<br>3.892	-11.3587<br>3.0283	4.34e-11<br>0.0036
**Cherp**	Calcium homeostasis endoplasmic reticulum protein	CHERP_MOUSE	MALE*	2.2514<br>1.3987	10.2957<br>9.4147	4.20e-09<br>2.38e-05
**Cldnd1**	Claudin domain-containing protein 1	CLDN1_MOUSE	FEMALE	1.4539	-9.4687	0.0434
**Cluh**	Clustered mitochondria protein homolog	CLU_MOUSE	MALE	1.6127	9.6364	0.0147
**Cnot3**	CCR4-NOT transcription complex subunit 3	CNOT3_MOUSE	MALE	1.106	9.1079	0.0001
**Col11a1**	Collagen alpha-1(XI) chain	COBA1_RAT	FEMALE	1.9882	-10.0225	0.0083
**Cog3**	Conserved oligomeric Golgi complex subunit 3	COG3_MOUSE	FEMALE<br>MALE	1.1847<br>0.1754	-5.9638<br>8.1053	0.0073<br>0.0441
**Col23a1**	Collagen alpha-1(XXIII) chain	CONA1_RAT	MALE	0.6327	8.6053	0.0091
**Cpne2**	Copine-2	CPNE2_MOUSE	FEMALE	0.6616	-8.6286	0.0055
**Crym**	Ketimine reductase mu-crystallin	CRYM_MOUSE	MALE	1.2699	9.2809	0.0395
**Ctnnal1**	Alpha-catulin	CTNL1_MOUSE	FEMALE	0.2623	-8.1917	0.0184
**Cit**	Citron Rho-interacting kinase	CTRO_MOUSE	MALE	2.1452	10.1862	0.0148
**Ddx3y**	ATP-dependent RNA helicase DDX3Y	DDX3Y_MOUSE	MALE*	4.0549<br>3.2276	12.1236<br>11.2887	6.34e-35<br>1.38e-22
**Dlk2**	Protein delta homolog 2	DLK2_MOUSE	MALE	0.1694	8.0967	0.0255
**Dmxl2**	DmX-like protein 2	DMXL2_MOUSE	FEMALE	0.5893	-8.5503	0.0064

Gene ID	Gene	Uniprot ID	Expression	Log_2CPM	Log_2FC	FDR
**Dync2h1**	Cytoplasmic dynein 2 heavy chain 1	DYHC2_RAT	MALE	0.8034	6.1817	0.0143
**Edc4**	Enhancer of mRNA-decapping protein 4	EDC4_MOUSE	MALE	2.6383	4.8642	0.0061
**Ahctf1**	Protein ELYS	ELYS_MOUSE	FEMALE	1.2382	-9.2431	0.0434
**Eno1**	Alpha-enolase	ENOA_RAT	FEMALE	5.2262	-1.6692	0.0123
**Eps15**	Epidermal growth factor receptor substrate 15	EPS15_MOUSE	FEMALE	2.6397	-10.6909	0.0103
**Erap1**	Endoplasmic reticulum aminopeptidase 1	ERAP1_RAT	MALE	2.0498	10.0889	9.27e-06
**Etfdh**	Electron transfer flavoprotein-ubiquinone oxidoreductase, mitochondrial	ETFD_MOUSE	FEMALE	1.6422	-9.6655	0.0195
**Ezh1**	Histone-lysine N-methyltransferase EZH1	EZH1_MOUSE	MALE	0.4115	8.3644	0.0143
**Fam126b**	Protein FAM126B	F126B_MOUSE	MALE	1.6117	7.8203	0.0147
**Fam83h**	Protein FAM83H	FA83H_MOUSE	MALE	0.5042	8.4641	0.0065
**Fastkd1**	FAST kinase domain-containing protein 1	FAKD1_MOUSE	FEMALE	0.9663	-7.0326	0.0024
**Fhl1**	Four and a half LIM domains protein 1	FHL1_RAT	FEMALE	2.2709	-10.3134	4.93e-06
**Flii**	Protein flightless-1 homolog	FLII_MOUSE	FEMALE	2.0696	-10.1073	0.0094
**Fuz**	Protein fuzzy homology	FUZZY_MOUSE	FEMALE	1.3708	-9.3818	1.00e-05
**Fzr1**	Fizzy-related protein homolog	FZR_MOUSE	MALE	0.2916	8.2326	0.0243
**Gata2**	Endothelial transcription factor GATA-2	GATA2_RAT	MALE	0.4306	6.5356	0.0335
**Slc25a22**	Mitochondrial glutamate carrier 1	GHC1_MOUSE	MALE	0.8264	8.8105	0.0036
**Gria3**	Glutamate receptor 3	GRIA3_MOUSE	MALE	3.1849	11.2452	0.0016
**Slc2a8**	Solute carrier family 2, facilitated glucose transporter member 8	GTR8_RAT	FEMALE	1.4321	-9.4458	7.50e-06
**Hcfc2**	Host cell factor 2	HCFC2_RAT	FEMALE	0.7009	-8.6703	0.0044
**Hdac5**	Histone deacetylase 5	HDAC5_CRIGR	MALE	1.4015	9.4178	5.90e-06

Gene ID	Gene	Uniprot ID	Expression	Log_2CPM	Log_2FC	FDR
**Hepacam**	Hepatocyte cell adhesion molecule	HECAM_MOUSE	MALE	0.2913	8.2318	0.0155
**Hes5**	Transcription factor HES-5	HES5_RAT	MALE	0.4392	8.3943	0.001
**Hipk2**	Homeodomain-interacting protein kinase 2	HIPK2_MESAU	FEMALE	0.2245	-8.1497	0.02
**Hsp90ab1**	Heat shock protein HSP 90-beta	HS90B_RAT	FEMALE	0.4423	-8.3901	0.0145
**Ift172**	Intraflagellar transport protein 172 homolog	IF172_MOUSE	MALE	0.6419	8.6126	0.0055
**Eif5**	Eukaryotic translation initiation factor 5	IF5_MOUSE	FEMALE	1.9854	-10.02	0.0067
**Ppa2**	Inorganic pyrophosphatase 2, mitochondrial	IPYR2_MOUSE	MALE	0.2085	8.1406	0.0236
**Kiaa0556**	Uncharacterized protein KIAA0556	K0556_MOUSE	FEMALE	1.2715	-9.2776	2.73e-05
**Kansl1l**	KAT8 regulatory NSL complex subunit 1-like protein	KAL1L_MOUSE	MALE	0.5851	8.5525	0.0044
**Kctd15**	BTB/POZ domain-containing protein KCTD15	KCD15_MOUSE	MALE	0.0662	7.9819	0.044
**Kcng4**	Potassium voltage-gated channel subfamily G member 4	KCNG4_MOUSE	MALE	2.0261	10.0649	4.52e-08
**Kdm1b**	Lysine-specific histone demethylase 1B	KDM1B_MOUSE	FEMALE	0.0825	-7.9911	0.0401
**Kdm5c**	Lysine-specific demethylase 5C	KDM5C_MOUSE	MALE	0.9258	8.9171	0.0006
**Kdm5d**	Lysine-specific demethylase 5D	KDM5D_MOUSE	MALE*	2.6350<br>2.3792	10.6875<br>10.3741	1.03e-13<br>2.19e-11
**Kdm6a**	Lysine-specific demethylase 6A	KDM6A_MOUSE	FEMALE<br>MALE*	1.6742<br>2.459<br>1.8701	-9.6982<br>10.5080<br>9.9039	2.53e-07<br>1.78e-12<br>2.40e-08
**Kif9**	Kinesin-like protein KIF9	KIF9_MOUSE	MALE	0.0779	7.9955	0.0407
**Krcc1**	Lysine-rich coiled-coil protein 1	KRCC1_MOUSE	FEMALE	1.7253	-9.7512	1.60e-06
**Rps6ka2**	Ribosomal protein S6 kinase alpha-2	KS6A2_MOUSE	MALE	0.8603	8.8476	0.0008
**Faim2**	Protein lifeguard 2	LFG2_RAT	FEMALE	1.4259	-9.4389	0.0254
**Lin7b**	Protein lin-7 homolog B	LIN7B_RAT	FEMALE*	1.1887<br>0.3157	-9.1904<br>-8.2504	4.51e-05<br>0.0173

Gene ID	Gene	Uniprot ID	Expression	Log_2CPM	Log_2FC	FDR
**L3mbtl3**	Lethal(3)malignant brain tumor-like protein 3	LMBL3_MOUSE	MALE	0.3534	8.3013	0.0189
**Aatk**	Serine/threonine-protein kinase LMTK1	LMTK1_MOUSE	MALE	0.7763	8.7569	0.0044
**Lrig2**	Leucine-rich repeats and immunoglobulin-like domains protein 2	LRIG2_MOUSE	MALE	2.0019	10.0396	3.73e-09
**Lrp3**	Low-density lipoprotein receptor-related protein 3	LRP3_RAT	MALE	0.3543	8.3007	0.0139
**Tmem57**	Macoilin	MACOI_MOUSE	MALE	1.5568	9.5797	3.06e-06
**Mamld1**	Mastermind-like domain-containing protein 1	MAMD1_MOUSE	MALE	0.8445	8.8306	0.0013
**Map6**	Microtubule-associated protein 6	MAP6_MOUSE	FEMALE	3.5744	-11.6383	0.0003
**Matk**	Megakaryocyte-associated tyrosine-protein kinase	MATK_MOUSE	FEMALE	0.5071	-8.4606	0.0099
**Mga**	MAX gene-associated protein	MGAP_MOUSE	MALE	2.0658	10.1059	0.0056
**Mgat5**	Alpha-1,6-mannosylglycoprotein 6-beta-N-acetylglucosaminyltransferase A	MGT5A_CRIGR	FEMALE	1.5922	-9.613	8.77e-07
**Mink1**	Misshapen-like kinase 1	MINK1_MOUSE	FEMALE	4.1235	-12.1920	6.33e-05
**Mapk13**	Mitogen-activated protein kinase 13	MK13_MOUSE	MALE	0.5931	8.5596	0.0094
**Mobp**	Myelin-associated oligodendrocyte basic protein	MOBP_MOUSE	MALE*	2.6728<br>0.9115	10.7258<br>8.9032	1.66e-10<br>0.0142
**Mpeg1**	Macrophage-expressed gene 1 protein	MPEG1_MOUSE	FEMALE	1.6734	-9.6972	0.0142
**Cdc42bpb**	Serine/threonine-protein kinase MRCK beta	MRCKB_MOUSE	MALE*	1.2818<br>4.8863	9.293<br>12.9594	0.0389<br>9.02e-45
**Mreg**	Melanoregulin	MREG_MOUSE	FEMALE	0.691	-8.6604	0.011
**Msl3**	Male-specific lethal 3 homolog	MS3L1_MOUSE	MALE	1.6052	9.6285	0.0173
**N4bp2l1**	NEDD4-binding protein 2-like 1	N42L1_MOUSE	MALE*	1.7175<br>1.2462	9.7463<br>9.2553	4.40e-07<br>2.99e-05
**Neurl4**	Neuralized-like protein 4	NEUL4_MOUSE	FEMALE	1.9839	-10.0185	1.70e-08
**Nfyc**	Nuclear transcription factor Y subunit gamma	NFYC_RAT	FEMALE	0.7236	-6.7245	0.021
**Olfm2**	Noelin-2	NOE2_RAT	FEMALE	2.7037	-10.7557	0.0132

Gene ID	Gene	Uniprot ID	Expression	Log_2CPM	Log_2FC	FDR
**Nrdc**	Nardilysin	NRDC_MOUSE	MALE	0.4011	8.3516	0.0112
**Nsun5**	Probable 28S rRNA (cytosine-C(5))-methyltransferase	NSUN5_MOUSE	MALE	1.3223	9.3338	0.0044
**Nudcd3**	NudC domain-containing protein 3	NUDC3_MOUSE	MALE	2.0976	10.138	4.20e-09
**Oma1**	Metalloendopeptidase OMA1, mitochondrial	OMA1_MOUSE	FEMALE	0.9356	-6.9995	0.0023
**Otof**	Otoferlin	OTOF_RAT	MALE	0.2185	8.1531	0.0335
**Pawr**	PRKC apoptosis WT1 regulator protein	PAWR_MOUSE	FEMALE	0.7591	-8.7334	0.0023
**Pcdhb14**	Protocadherin beta-14	PCDBE_MOUSE	FEMALE	1.3663	-9.3771	7.89e-06
**Pcnx**	Pecanex-like protein 1	PCX1_MOUSE	MALE	2.2225	5.6478	1.99e-05
**Per3**	Period circadian protein homolog 3	PER3_RAT	FEMALE	2.2536	-8.3834	0.0094
**Rabggta**	Geranylgeranyl transferase type-2 subunit alpha	PGTA_RAT	MALE	0.5895	8.5586	0.01
**Phyhip**	Phytanoyl-CoA hydroxylase-interacting protein	PHYIP_RAT	FEMALE	2.5971	-10.6471	2.02e-12
**Pitpna**	Phosphatidylinositol transfer protein alpha isoform	PIPNA_MOUSE	FEMALE<br>MALE	2.0014<br>2.1982	-8.1146<br>5.7933	6.06e-08<br>0.0003
**Plec**	Plectin	PLEC_CRIGR	MALE	1.7266	9.7561	0.0125
**Plod3**	Procollagen-lysine,2-oxoglutarate 5-dioxygenase 3	PLOD3_MOUSE	MALE	0.543	8.5086	0.0141
**Plxnb2**	Plexin-B2	PLXB2_MOUSE	MALE	2.7341	10.7881	6.75e-15
**Ppp1r3e**	Protein phosphatase 1 regulatory subunit 3E	PPR3E_MOUSE	MALE	0.1543	8.0792	0.0436
**Prex2**	Phosphatidylinositol 3,4,5-triphosphate-dependent Rac exchanger 2 protein	PREX2_MOUSE	MALE	1.7503	4.3581	0.0205
**Primpol**	DNA-directed primase/polymerase protein	PRIPO_MOUSE	MALE	0.3368	8.2826	0.0143
**Prkra**	Interferon-inducible double-stranded RNA-dependent protein kinase activator A	PRKRA_RAT	MALE	1.4564	9.4753	0.024
**Ptprn**	Receptor-type tyrosine-protein phosphatase-like N	PTPRN_RAT	MALE	1.8027	9.8332	0.0094
**Ptpro**	Receptor-type tyrosine-protein phosphatase O	PTPRO_MOUSE	MALE	0.0993	8.0179	0.0458

Gene ID	Gene	Uniprot ID	Expression	Log_2CPM	Log_2FC	FDR
**Pus7l**	Pseudouridylate synthase 7 homolog-like protein	PUS7L_MOUSE	FEMALE	0.3236	-8.2596	0.0165
**Rb1cc1**	RB1-inducible coiled-coil protein 1	RBCC1_MOUSE	MALE	0.5686	8.5351	0.0069
**Rbm45**	RNA-binding protein 45	RBM45_RAT	FEMALE	1.7605	-9.7882	0.0173
**Rexo1**	RNA exonuclease 1 homolog	REXO1_MOUSE	MALE	0.2996	8.2417	0.0165
**Rfx5**	DNA-binding protein Rfx5	RFX5_MOUSE	FEMALE<br>MALE*	8.3022<br>4.1427<br>3.1396	-7.1923<br>1.8574<br>2.3186	2.60e-25<br>0.0061<br>0.0123
**Rgs8**	Regulator of G-protein signaling 8	RGS8_RAT	MALE	5.2094	6.2431	1.02e-09
**Riok1**	Serine/threonine-protein kinase RIO1	RIOK1_MOUSE	MALE	0.1161	8.0368	0.0363
**Rnf212**	Probable E3 SUMO-protein ligase RNF212	RN212_MOUSE	MALE	0.6076	5.9705	0.0401
**Rapgef2**	Rap guanine nucleotide exchange factor 2	RPGF2_MOUSE	MALE	1.4172	9.434	7.59e-06
**Rreb1**	Ras-responsive element-binding protein 1	RREB1_MOUSE	MALE	0.9079	8.8995	0.0034
**Rtf1**	RNA polymerase-associated protein RTF1 homolog	RTF1_MOUSE	FEMALE	1.9768	-10.0117	0.012
**Rubcn**	Run domain Beclin-1-interacting and cysteine-rich domain containing protein	RUBIC_MOUSE	FEMALE	1.2494	-5.6551	0.0088
**Slc12a6**	Solute carrier family 12 member 6	S12A6_MOUSE	FEMALE	2.0197	-10.0551	0.0062
**Sec61a1**	Protein transport protein Sec61 subunit alpha isoform 1	S61A1_RAT	MALE	0.5554	8.5207	0.0085
**Sec22c**	Vesicle-trafficking protein SEC22c	SC22C_MOUSE	FEMALE	0.731	-8.7037	0.0123
**Sdccag3**	Serologically defined colon cancer antigen 3 homolog	SDCG3_MOUSE	MALE	1.2292	9.2385	0.0437
**Setd5**	SET domain-containing protein 5	SETD5_MOUSE	MALE	2.3497	10.3963	5.75e-12
**St6galnac4**	Alpha-N-acetyl-neuraminyl-2,3-beta-galactosyl-1,3-N-acetyl-galactosaminide alpha-2,6-sialyltransferase	SIA7D_MOUSE	MALE	0.7586	8.7371	0.0125
**Snx24**	Sorting nexin-24	SNX24_RAT	FEMALE	0.0997	-8.0101	0.0361
**Spata7**	Spermatogenesis-associated protein 7 homolog	SPAT7_MOUSE	MALE	1.7324	7.9474	0.031

Gene ID	Gene	Uniprot ID	Expression	Log_2CPM	Log_2FC	FDR
**Stra6**	Stimulated by retinoic acid gene 6 protein homolog	STRA6_RAT	FEMALE	0.4312	-8.3769	0.022
**Suco**	SUN domain-containing ossification factor	SUCO_MOUSE	MALE	1.3462	9.36	9.65e-06
**Sympk**	Symplekin	SYMPK_MOUSE	MALE	1.3501	5.3435	0.0441
**Rars**	Arginine--tRNA ligase, cytoplasmic	SYRC_CRIGR	FEMALE	2.7887	-10.8424	0.0011
**Tll1**	Tolloid-like protein 1	TLL1_MOUSE	FEMALE<br>MALE	1.0197<br>0.3631	-6.2939<br>8.3103	0.0061<br>0.0125
**Tmem18**	Transmembrane protein 18	TMM18_RAT	FEMALE	2.0363	-10.0726	6.31e-09
**Txnrd3**	Thioredoxin reductase 3	TRXR3_MOUSE	FEMALE	0.4357	-8.383	0.0139
**Txndc11**	Thioredoxin domain-containing protein 11	TXD11_MOUSE	FEMALE	1.5653	-7.6703	0.0044
**Tyk2**	Non-receptor tyrosine-protein kinase TYK2	TYK2_MOUSE	FEMALE	1.5463	-9.5649	0.0193
**Usp14**	Ubiquitin carboxyl-terminal hydrolase 14	UBP14_MOUSE	MALE	0.8542	8.8392	0.0119
**Usp16**	Ubiquitin carboxyl-terminal hydrolase 16	UBP16_RAT	MALE	0.4519	8.4082	0.0098
**Unc13a**	Protein unc-13 homolog A	UN13A_MOUSE	MALE	2.0976	5.732	0.0026
**Usp9x**	Probable ubiquitin carboxyl-terminal hydrolase FAF-X	USP9X_MOUSE	MALE*	3.6714<br>3.1267	11.7372<br>11.1865	5.86e-27<br>1.48e-18
**Uty**	Histone demethylase UTY	UTY_MOUSE	MALE*	2.8309<br>1.6003	10.8864<br>9.6247	1.26e-16<br>8.76e-07
**Vmn2r116**	Vomeronasal type-2 receptor 116	V2116_MOUSE	FEMALE	0.3412	-8.2786	0.0143
**Vasp**	Vasodilator-stimulated phosphoprotein	VASP_MOUSE	FEMALE	0.2785	-8.2087	0.0401
**Hdlbp**	Vigilin	VIGLN_MOUSE	MALE	0.2214	8.1544	0.0203
**Vps13c**	Vacuolar protein sorting-associated protein	VP13C_MOUSE	MALE	4.0828	8.1066	4.31e-21
**Wdfy3**	WD repeat and FYVE domain-containing protein 3	WDFY3_MOUSE	MALE	1.3295	6.2107	0.0045
**Wiz**	Protein Wiz	WIZ_MOUSE	FEMALE	2.1174	-10.156	1.11e-08
**Wnk2**	Serine/threonine-protein kinase WNK2	WNK2_MOUSE	MALE	1.7661	9.797	4.56e-06

Gene ID	Gene	Uniprot ID	Expression	Log_2CPM	Log_2FC	FDR
**Xpo4**	Exportin-4	XPO4_MOUSE	FEMALE	1.2088	-9.2121	0.0458
**Yme1l1**	ATP-dependent zinc metalloprotease YME1L1	YMEL1_MOUSE	MALE	0.8076	5.6701	0.0193
**Zbtb46**	Zinc finger and BTB domain-containing protein 46	ZBT46_MOUSE	MALE	0.6018	8.5702	0.0038
**Zfyve16**	Zinc finger FYVE domain-containing protein 16	ZFY16_MOUSE	FEMALE	0.4596	-8.409	0.0193
**Znf569**	Zinc finger protein 569	ZN569_MOUSE	FEMALE	0.4599	-8.4091	0.0077
**Znf18**	Zinc finger protein 18	ZNF18_RAT	FEMALE<br>MALE	-4.8543<br>2.0655	3.2728<br>3.8641	0.0031<br>0.0293
**Zswim6**	Zinc finger SWIM domain-containing protein 6	ZSWM6_MOUSE	MALE	2.5167	10.5679	0.0044

Supplemental Table S2 Gene ontology enrichment analysis
A comprehensive list of the 142 gene ontology (GO) terms that were enriched in male hamster brains compared with female hamster brains.
(BP = biological process; CC = cellular component; MF = molecular function; FDR = false discovery rate)

GO term	Ontology	Over-represented FDR
Cardiac muscle cell contraction	BP	7.52E-09
Cerebellar cortex structural organization	BP	0
T cell antigen processing and presentation	BP	2.09E-07
Regulation of forebrain neuron differentiation	BP	0.0236
Notochord morphogenesis	BP	0.001
Mesodermal cell differentiation	BP	0.001
Somite rostral/caudal axis specification	BP	0.0005
Histone H3-K27 demethylation	BP	0.0011
Histone H3-K4 demethylation	BP	4.49E-06
Respiratory system process	BP	0.001
Actin-mediated cell contraction	BP	7.58E-06
Cardiovascular system development	BP	0.003
Histone H3-K36 demethylation	BP	4.49E-06
Anatomical structure arrangement	BP	7.09E-08
Histone lysine demethylation	BP	8.82E-13

GO term	Ontology	Over-represented FDR
Histone demethylation	BP	8.82E-13
Protein demethylation	BP	8.82E-13
Protein dealkylation	BP	8.82E-13
Regulation of chromatin silencing	BP	3.13E-05
Translational initiation	BP	7.09E-08
Demethylation	BP	1.69E-11
BMP signaling pathway	BP	1.99E-07
Chromosome segregation	BP	4.51E-15
Axon extension	BP	1.03E-05
Anterior/posterior axis specification	BP	0.0152
Respiratory gaseous exchange	BP	0.0227
Embryonic axis specification	BP	0.0273
Regulation of gene silencing	BP	0.0006
Neural tube development	BP	0.0004
Actin filament-based movement	BP	0.0008
RNA secondary structure unwinding	BP	7.52E-06
Cellular response to transforming growth factor beta stimulus	BP	0.0001
Heart morphogenesis	BP	0.0006
Transforming growth factor beta receptor signaling pathway	BP	1.75E-05
Neuron projection extension	BP	0.0001
Response to transforming growth factor beta	BP	0.0002
Antigen processing and presentation	BP	0.0008
Canonical Wnt signaling pathway	BP	0.0011
Hippocampus development	BP	0.0003
Post-embryonic development	BP	0.001
Neuron migration	BP	5.44E-05
Developmental cell growth	BP	0.0009
Transmembrane receptor protein serine/threonine kinase signaling pathway	BP	0.0003
Protein deubiquitination	BP	0.0007
Developmental growth involved in morphogenesis	BP	0.003

GO term	Ontology	Over-represented FDR
Cell growth	BP	0.0009
Chordate embryonic development	BP	5.63E-08
Embryo development ending in birth or egg hatching	BP	5.76E-08
In utero embryonic development	BP	2.81E-07
Heart development	BP	7.53E-06
Embryonic organ development	BP	0.008
Embryo development	BP	1.99E-07
Protein modification by small protein removal	BP	0.002
Epithelium development	BP	0.0092
Mitotic nuclear division	BP	0.0004
Tube development	BP	0.0361
Developmental growth	BP	0.0115
Histone modification	BP	9.25E-06
Cellular response to growth factor stimulus	BP	0.0073
Growth	BP	0.0002
Covalent chromatin modification	BP	1.06E-05
Nuclear division	BP	0.0027
Response to growth factor	BP	0.0144
Organelle fission	BP	0.0045
Chromatin modification	BP	2.61E-05
Ubiquitin-dependent protein catabolic process	BP	0.0162
Modification-dependent protein catabolic process	BP	0.0178
Chromatin organization	BP	6.61E-05
Modification-dependent macromolecule catabolic process	BP	0.02
Organ morphogenesis	BP	0.0408
Proteolysis involved in cellular protein catabolic process	BP	0.0114
Cell division	BP	0.0197
Proteolysis	BP	0.0375
Movement of cell or subcellular component	BP	0.0139
Cellular protein modification process	BP	5.56E-06

GO term	Ontology	Over-represented FDR
Protein modification process	BP	5.56E-06
Macromolecule modification	BP	2.16E-05
Single-organism organelle organization	BP	0.0023
Cellular protein metabolic process	BP	4.97E-05
Protein metabolic process	BP	4.83E-06
Anatomical structure development	BP	0.0067
Regulation of gene expression	BP	9.61E-05
Organelle organization	BP	0.0064
Regulation of transcription, DNA-templated	BP	0.0061
Regulation of nucleic acid-templated transcription	BP	0.0065
Regulation of RNA biosynthetic process	BP	0.0065
Regulation of RNA metabolic process	BP	0.0142
Regulation of macromolecule biosynthetic process	BP	0.0341
Regulation of cellular macromolecule biosynthetic process	BP	0.0441
Regulation of macromolecule metabolic process	BP	0.0012
Regulation of biosynthetic process	BP	0.0462
Macromolecule metabolic process	BP	1.95E-06
Cellular macromolecule metabolic process	BP	4.16E-05
Cellular component organization	BP	0.0176
Cellular component organization or biogenesis	BP	0.0217
Regulation of metabolic process	BP	0.0115
Primary metabolic process	BP	0.0006
Organic substance metabolic process	BP	0.0024
Metabolic process	BP	0.005
Cellular metabolic process	BP	0.0309
Apical part of cell	CC	2.47E-06
Cytoplasmic ribonucleoprotein granule	CC	0.0001
Ribonucleoprotein granule	CC	0.0002
Histone methyltransferase complex	CC	0.0335
Methyltransferase complex	CC	0.0491

GO term	Ontology	Over-represented FDR
Growth cone	CC	0.02
Site of polarized growth	CC	0.0234
Co-SMAD binding	MF	0
Histone demethylase activity (H3-K27 specific)	MF	2.78E-08
Histone demethylase activity (H3-K36 specific)	MF	1.27E-06
Histone demethylase activity (H3-K4 specific)	MF	2.66E-06
Histone demethylase activity	MF	3.71E-13
Demethylase activity	MF	8.82E-13
Oxidoreductase activity, acting on paired donors, with incorporation or reduction of molecular oxygen, 2-oxoglutarate as one donor, and incorporation of one atom each of oxygen into both donors	MF	1.72E-12
SMAD binding	MF	2.63E-06
Dioxygenase activity	MF	7.36E-11
Oxidoreductase activity, acting on paired donors, with incorporation or reduction of molecular oxygen	MF	1.28E-08
ATP-dependent RNA helicase activity	MF	0.0005
RNA-dependent atpase activity	MF	0.0005
RNA helicase activity	MF	0.0006
Ubiquitinyl hydrolase activity	MF	0.0003
ATP-dependent helicase activity	MF	0.001
Purine NTP-dependent helicase activity	MF	0.001
Cysteine-type peptidase activity	MF	0.0041
Helicase activity	MF	0.003
RNA polymerase II core promoter proximal region sequence-specific DNA binding	MF	0.0225
Core promoter proximal region sequence-specific DNA binding	MF	0.0264
Core promoter proximal region DNA binding	MF	0.028
Atpase activity, coupled	MF	0.005
RNA polymerase II regulatory region sequence-specific DNA binding	MF	0.0399
RNA polymerase II regulatory region DNA binding	MF	0.0491
Atpase activity	MF	0.0028
Peptidase activity, acting on L-amino acid peptides	MF	0.0176

GO term	Ontology	Over-represented FDR
Oxidoreductase activity	MF	0.0045
Peptidase activity	MF	0.02
Pyrophosphatase activity	MF	0.0273
Hydrolase activity, acting on acid anhydrides, in phosphorus-containing anhydrides	MF	0.0275
Hydrolase activity, acting on acid anhydrides	MF	0.0278
Nucleoside-triphosphatase activity	MF	0.0441
Hydrolase activity	MF	0.047
Catalytic activity	MF	0.0014
